# Hospital nurses’ knowledge regarding older patients: a multicenter study

**DOI:** 10.1186/s12912-021-00604-4

**Published:** 2021-08-04

**Authors:** Christel T. A. J. Derks, Marjo M. G. M. Hutten - van den Elsen, Lysette J. Hakvoort, Mariëlle P. J. van Mersbergen, RENurse Consortium, Marieke J. Schuurmans, Jeroen Dikken

**Affiliations:** 1 Elisabeth Tweesteden Ziekenhuis, Tilburg, the Netherlands; 2 Deventer Ziekenhuis, Deventer, the Netherlands; 3 Máxima Medisch Centrum, Eindhoven, the Netherlands; 4 Rijnstate, Arnhem, the Netherlands; 5 Martini Ziekenhuis, Groningen, the Netherlands; 6 Franciscus Gasthuis & Vlietland, Rotterdam, the Netherlands; 7 Isala, Zwolle, the Netherlands; 8 Medisch Spectrum Twente, Enschede, the Netherlands; 9 Spaarne Gasthuis, Hoofddorp, the Netherlands; 10 Albert Schweitzer ziekenhuis, Dordrecht, the Netherlands; 11 Nursing Science, Julius Center University Medical Center/University Utrecht, Utrecht, the Netherlands; 12 Faculty of Health, Nutrition & Sport & Health Innovation Centre of Expertise, The Hague University of Applied Sciences, The Hague, the Netherlands

**Keywords:** KOP-Q, Knowledge, Attitude, Older patients, Geriatrics, Registered nurses, The Netherlands

## Abstract

**Background:**

Nursing care in hospitals increasingly involves older adults. A nursing workforce able to care for the ageing population is therefore critical for ensuring quality older adult care. Gaining insight in the knowledge and attitudes of nurses regarding older patients in the Netherlands is needed to develop and increase the impact of education- and quality improvement programs which can positively influence nurses’ knowledge and attitudes regarding older patients.

**Methods:**

A cross-sectional multicenter study was performed. Data was collected in ten tertiary medical teaching hospitals well spread across the Netherlands (89 wards, 2902 nurses). Knowledge levels were measured using the Knowledge about Older Patient-Quiz (KOP-Q), consisting of 30 true-false questions. Knowledge levels of registered nurses are compared with knowledge levels known from literature of first year nursing students; last year nursing students; nurses; and nurse specialist. Potential associated factors considered were: age; sex; education; experience; opinions and preferences. Opinion and preferences regarding working with older patients were measured by three questions: 1) which patient group nurses preferred to work with; 2) how nurses feel about the increase of older patients in the hospital; and 3) whether nurses find it difficult to care for older patients.

**Results:**

From all wards, a representative sample of 1743 registered hospital nurses working on all 89 wards participated. On all wards, a large range in knowledge levels is observed between nurses, with 37% of nurses presenting knowledge levels comparable with nursing student and 31% of nurses presenting knowledge levels comparable with nurse specialists. Knowledge is related to age (*p* < .001), work experiences (*p* < .001), preparatory secondary education (*p* < .001) and nurses education level (*p* = .012). A minority (12.5%) prefers working with older patients and most nurses do not find it difficult.

**Conclusions:**

This study shows that there is a large diversity in knowledge levels of Dutch hospital nurses in every hospital, on every ward. A majority of nurses demonstrate negative opinions and preferences. This implies that older patients admitted can receive different levels of quality of care on the same day as nurses with different knowledge levels provide care during the various shifts. Findings demonstrate an urgent need for education programs with themes regarding essential care for older patients in the Netherlands.

**Supplementary Information:**

The online version contains supplementary material available at 10.1186/s12912-021-00604-4.

## Background

In the Netherlands, the older population (aged 65 and over) accounted for 19.2% of the population in 2019, and is predicted to be 25.5% by the year 2040 [1]. Because of these demographic changes, nursing care in hospitals increasingly involve older patients [2]. Older people are more likely to experience multiple chronic health conditions, have issues related to poly-pharmacy and often require additional support with activities of daily living [3]. Furthermore, the complexity of care needs of older people has increased as a result of life prolonging advancements in healthcare [4]. This results in a rising demand for nurses demonstrating excellent knowledge, attitudes and skills regarding the care for older patients as nurses are directly influencing the quality of care older patients receive [5].

Several studies have investigated the knowledge and attitudes of nurses towards older patients [4, 6–9]. These studies found that care for older people is not considered a very attractive area of nursing practice [4, 7, 8, 10], mostly as a result of professional disrespect for choosing to work with older people and the related low status of older people in the hospital setting [11, 12]. Older patients can be considered a burden and obstacle to the more important work of caring for younger adults, with some nurses finding care for cognitively impaired older people difficult and frustrating [13]. Rush et al. [9] found that nurses’ held coexisting positive and negative attitudes towards generic and specific aspects of older adult care. Negative attitudes, in particular, were directed at the characteristics of older adults, their care demands or where reflected in nurses’ approaches to care.

Many of the studies measuring nurses’ knowledge and attitudes included by Liu et al. [7] and Rush et al. [9] had methodological limitations and both highlight the paucity of research examining nurses’ knowledge and attitudes towards older adult care. One of the most important limitation was the poor quality of measurement instruments used which was already noticed in the year 2000 [6], but no new instruments were adequately developed and validated between 2000 and 2013. Thereafter, Dikken et al. developed and validated a new instrument, the Knowledge about Older Patients – Quiz (KOP-Q) [14, 15] in order to investigate the knowledge levels about older patients among nursing student and hospital nurses [16]. The results from this study were in line with previous results from studies included by Courtney et al. [6], Liu et al. [7] and Rush et al. [9]: a substantial proportion of participants in all groups demonstrated insufficient knowledge about older patients. Because of methodological limitations encountered in previous studies [6, 7], and a small number of participating hospitals and nurses included in the more recent studies regarding nurses’ knowledge levels [16], the generalizability of results for the Netherlands can be questioned.

The aim of this study was, therefore, to explore the current knowledge of hospital nurses in the Netherlands regarding older patients using a representative sample size. Furthermore, it was tested whether the level of knowledge is associated with age, level of education, working experience and specialty of ward. Finally, three additional questions regarding nurses’ opinions and preferences towards working with older patients are explored in relation with knowledge levels. The analysis were guided by four research questions:

1. What are the current knowledge levels of hospital nurses in the Netherlands?
2. What factors are associated with the knowledge levels of nurses in the Netherlands?
3. What are nurses’ opinions and preferences regarding care for older patients?
4. How are opinions and preferences related with knowledge levels demonstrated by nurses in the Netherlands?

As there is a paucity of research examining nurses attitudes and knowledge towards older patients, results from this large multicenter study can help hospitals in prioritizing and highlighting the importance of educational and/or quality improvement programs regarding care for older patients in the future.

## Methods

### Design

This study used a multicenter cross-sectional design.

### Setting, participants and data collection

Participants were recruited from ten tertiary medical teaching hospitals in the Netherlands, which are affiliated with the Research and Education in Nursing (RENurse) consortium. Hospitals are located in both urban and rural areas across the Netherlands. The research coordinator of each participating hospital approached managers from different wards where older patients are admitted. Each hospital included at least two surgical wards and two internal medicine wards. Included wards were regular wards (e.g. surgical ward, internal medicine ward, lung medicine ward, etc), daycare surgery, intensive care, emergency room and wards specialized in dialysis. When the managers gave consent for participation, all nurses who met the inclusion criteria received an e-mail with information about the research and a link to the online survey. The online survey consisted of the information letter and an informed consent page, followed by the Knowledge about Older Patient-Quiz (KOP-Q) [14, 15]. The online survey was developed using SurveyMonkey which is online survey software to create and run professional online surveys [17]. Inclusion criteria for participants in this study were: 1) being a registered nurse, 2) having a main contract on one ward, 3) providing informed consent. Student nurses were excluded for participating in this study.

Data were collected between February 1st 2018 and March 8th 2018. Using a margin of error of 5% and a confidence level of 99.9%, a minimum of 1068 respondents was needed to be representative for the total number of hospital nurses working in the Netherlands (*n* = 77.175 in 2018, [18]). Of the 2902 registered nurses who were invited to participate, a total of 1922 completed the questionnaire (66.2%). Participants, who did not provide informed consent, were excluded from the data analysis (*n* = 21). Participants, who did not meet the other inclusion criteria were also excluded (*n* = 6). Finally, 152 participants did not complete all the questions of the KOP-Q and were, therefore, excluded. This led to a total sample of 1743 registered nurses. In all participating hospitals, the overall response rate of included participants reached a minimum of 60% and registered nurses participating were highly representative for the nursing population in Dutch hospitals.

### The knowledge about older patients-quiz

The level of knowledge was measured by the KOP-Q instrument [14, 15]. The KOP-Q contains 30 dichotomous items (true/false) measuring general knowledge regarding older hospitalized patients regarding six themes: normal aging, geriatric conditions, signaling problems with old age, interventions, family interventions and vulnerable patients versus older patients [14], with every correct answer assigned 1 point and every incorrect answer 0 points [15]. The KOP-Q was previously developed and validated for the Netherlands and demonstrated adequate face validity, good readability [14], a good scale content Validity Index/average (S-CVI/ave. = .91) [15]. Psychometric validity of the KOP-Q was previously assessed using Item Response Theory [15], looking at the discrimination and difficulty parameters. Most KOP-Q items had moderate to high discrimination values (indicating to what extent the item is good in discriminating between knowledgeable and less-knowledgeable respondents [19]). The range at which the KOP-Q retrieves information about knowledge level of participants (difficulty) is *β* − 10.2 to 0.7, indicating that most items are easy to answer even if knowledge levels are low [19]. Finally, the reliability for all knowledge items was considered good (internal consistency by Kuder-Richardson Formula 20 = .70) [15]. The KOP-Q is also cross-culturally validated for use in the United States of America [20].

The maximum score of the KOP-Q is 30, the minimum is 0. For easy interpretation of test scores, and compare individual and group scores, Dikken er al. presented normative data for four known groups: 1) first years bachelor of nursing students, 2) final year bachelor of nursing students, 3) registered nurses and 4) nurse specialist [15]. This study also presented threshold scores between first- and final year students (21.09 points), final year students and registered nurses (24.25 points), and the threshold between registered nurses and nurse specialists (26.77 points), with threshold scores representing the scores at which an individual shifts to a more (or less) knowledgeable group [15]. We used these previous presented known group threshold scores for interpreting results of the registered nurses included in this study.

### Related variables

Knowledge is closely related with attitude constructs such as opinion and preference regarding working with older patients [21, 22], and therefore three additional questions were formulated by the research group. Firstly, nurses were asked which patient age category they preferred to work with (age 0-18, 19-69, 70+). Secondly, nurses were asked how they feel about the increase of older patients in the hospital (indicated on a scale from 1, no problem et al., to 10, a major problem). Finally, nurses were asked whether they find it difficult to care for older patients (indicated on a scale from 1, very easy to 10, very difficult). Additional demographic information known from the literature being potentially contributing factors associated with nurses attitudes towards gerontology care, such as age, level of education and years of working experiences and type of ward were also collected [23].

### Statistical analyses

First the knowledge variable was tested for normality for total KOP-Q scores, which was confirmed. Then, for the included sample, sum scores on the KOP-Q were classified in four groups corresponding with the four known groups described in the literature to interpreted results of the KOP-Q (i.e. first years bachelor of nursing students, final years bachelor of nursing students, registered nurses and nurse specialists) [15]. To find possible associated variables of knowledge, assumptions of linearity were checked for the related variables. If there was no linearity, dummy variables were made for the independent variables. After testing possible associated variables for multicollinearity, multiple regression analysis was performed to test the influence of the factors: age; educational level secondary school; educational level nursing school; years of working experience; and type of ward (surgical or internal medicine) on the level of knowledge. This result was significant at the *p* ≤ .05 level. Age and years of work experiences proved to have multicollinearity (r = .917) and were, therefore, assessed separately in the final model.

Finally, a hypothesis was formulated that knowledge was positively correlated with attitude constructs “opinion” and “preferences”. The Pearson correlation test was used to test this hypothesis. Differences in preferences between groups was tested with independent sample *t*-test. Data analysis was performed using Statistical Packages for the Social Sciences (SPSS), version 24.0 [24].

### Ethical considerations

The procedures of this study were reviewed and approved by METC Brabant (the medical ethics committee), METC protocol number NW-2017-08. All participating hospitals provided formal approval for this study. All nurses who met the inclusion criteria received an e-mail with information about the research (information letter) and a link to the online survey. The first page of the online survey was again the information letter and an informed consent page. Only after providing informed consent, participants proceeded to the online survey. If consent was not given, the online survey was closed and no responses were collected.

## Results

### Sample characteristics

Registered nurses included in this study (*n* = 1743) where mostly female (91,5%) and had a mean of 15.81 (SD. 12.3) years of experience. 34.8% of nurses had an educational nursing background on the vocational level, 35.1% on the bachelor level and 27.5% was in-service educated (Table 1). The name “In-service education” is used in the Netherlands for education developed and provided before 1997 by hospitals to train their own nurses in an employee-student position. In-service education took 4 years to complete. From 1997, in-service education was replaced by two main types of nursing education in schools: one on the vocational level- and one at the bachelor level of nursing, both also taking 4 years to complete.

**Table 1:** Characteristics of participants with no missing values on the Knowledge about Older Patients-Quiz (*n* = 1743)

**Characteristics**	
Sex, female, n (%)	1590 (91.5%)
*Missing, n*	*5* (0.3%)
Age, mean (SD)	38.7 (12.4)
*Missing, n*	*122* (7.0%)
**Level of high school education** (%)	
Preparatory secondary vocational education (4 years)	876 (50.3%)
Preparatory secondary higher education (5 years)	686 (39.4%)
Pre-university education (6 years)	108 (6.2%)
*Missing, n*	*73* (4.2%)
**Level of nursing education, n(%)**	
Vocational	607 (34.8%)
Bachelor	611 (35.1%)
In-service	479 (27.5%)
*Missing, n*	*46* (2.6%)
**Years of experience, mean (SD)**	15.81 (12.3)
*Missing, n (%)*	*25* (1.4%)
**Type of ward where nurses currently working, n (%)**	
Internal medicine	910 (52.2%)
Surgery specialties	597 (34.3%)
Combinations ward with internal medicine and surgery specialties	220 (12.6%)
Unknown	3 (0.2%)
Different	13 (0.7%)
*Missing, n*	0 (0%)

### Knowledge of Dutch nurses

**Fig. 1:**
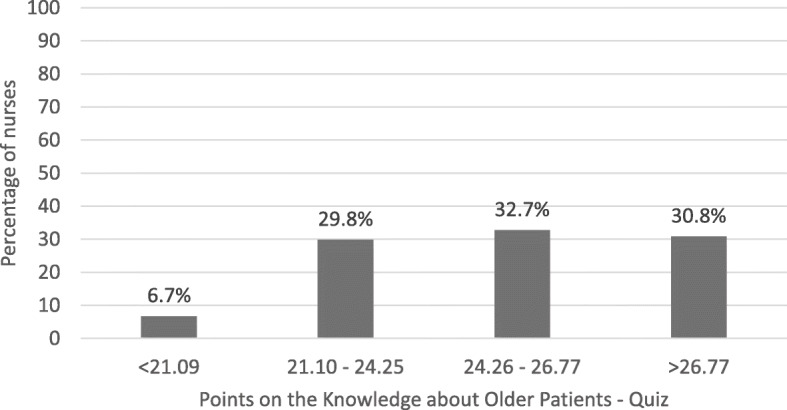
Percentage of nurses (*n* = 1743) demonstrating knowledge levels within the four known groups as described by Dikken et al., 2016 [15]

Figure 1 presents the percentage of registered nurses with knowledge levels comparable with the four known groups as presented in a previous study by Dikken et al. [15]. Of the registered nurses in this study, 116 nurses (6.7%) demonstrated knowledge levels comparable with first year student knowledge levels (scoring < 21.09 points) and 519 nurses (29.8%) having knowledge levels comparable with final year student knowledge levels (scoring between 21.10 – 24.25 points). A total of 571 nurses (32.7%) demonstrated knowledge levels comparable with registered nurses (scoring between 24.26 – 26.77 points) and 537 nurses (30.8%) had knowledge levels comparable with nurse specialist knowledge levels (scoring> 26.77 points). A wide range in knowledge levels was observed between nurses in all hospitals and on all wards (Additional file 1). The range of knowledge levels of registered nurses varied with a minimum score of 15 points (*n* = 3) to a maximum of 30 points (*n* = 23), meaning a difference of 15 points on the KOP-Q (i.e. 15 correct answers).

In Table 2, all items with less than 90% of nurses answering the KOP-Q item correct are presented (*n* = 12 items). When looking at the content of these items, themes with the highest percentage of incorrect answers regarded cognitive problems (i.e. depression, delirium, dementia), medication, use of hearing aids, risk of falling and stress-incontinence. For an overview of the percentages correct and incorrect of all KOP-Q items, see Additional file 2.

**Table 2:** Items of the KOP-Q with a percentage of correct answered < 90%

Item nr.	Question of the Knowledge about Older Patient - Quiz	Percentage correct	Percentage wrong
1	Forgetfulness, concentration issues, and indecisiveness are parts of aging rather than indicators of depression.	53.2%	46.8%
5	In general, older people are more sensitive to medication because their kidney and liver functions are declining.	86.1%	13.9%
8	Patients rarely remember that they were anxious and/or restless during delirium.	67.8%	32.2%
13	Lowering the frequency of a medication is an effective intervention to achieve (medication) adherence by patients.	79.8%	20.2%
19	It is good to provide extensive instruction about how to complete tasks to patients with apraxia.	58.2%	41.8%
20	When speaking to hearing-impaired older patients, it is best to speak at normal volume.	56.7%	43.3%
22	In the case of difficulty swallowing, all medicines must be ground to ensure that patients ingest them.	72.2%	27.8%
25	As a nurse, you have to speak clearly into the ear of hearing-impaired older patients.	61.7%	38.3%
26	Pain medication should be administered to older people as little as possible, due to the possibility of addiction.	86.9%	13.1%
16	In the case of delirium, bright lighting should be used to illuminate all of the corners of the room.	70.3%	29.7%
29	The risk of falling is higher for people in the hospital setting than in those who are living at home.	65.3%	34.7%
30	Stress incontinence may occur in patients who are not capable of opening their own trousers.	62.9%	37.1%

A statistically significant association was found between higher knowledge levels and age and work experiences, with older, more experiences nurses demonstrating higher knowledge levels (*p* < .001). Furthermore, higher level of preparatory secondary education (*p* < .001) and a higher level of nursing education were also associated to higher knowledge levels (*p* = .012). No statistically significant association was found between knowledge levels and the type of ward where nurses are working (*p* = .326).

### Knowledge in relation with opinions and preferences

Of the registered nurses, 12.5% (*n* = 209) answered the question “which target group would you prefer to work with” positively regarding the care for older patients. The majority of them (*n* = 1294, 77.6%) preferred to care for middle-aged patients and 164 (9.8%) nurses preferred to care for children, even though nurses working in pediatric wards were excluded from participation in this study. Table 3 shows that participants who like to work with older patients (70+) have significantly higher knowledge levels compared with nurses who prefer to work with patients aged 0-18 (*p* < .001) or patients aged 19-69 (*p* = .006). Another result was that most nurses do not find it difficult to take care of older patients (means for the groups were 3.65, 3.65 and 2.70 accordingly on a scale from 1, very easy to 10, very difficult). Furthermore, nurses who do not prefer taking care of older patients (70+), think the increase of older patients in the hospitals is a greater problem compared with nurses preferring to work with older patients (*p* < .001). Finally, they find it more difficult to take care of them compared with nurses who prefer to work with older patients (*p* < .001).

**Table 3:** Knowledge in relation with opinions and preferences

	Which target group would you prefer to work with?		
	0-18 (***n*** = 164)	19-69(***n*** = 1294)	70 + (***n*** = 209)
**What do you think of an increase in the number of older people? (Scale 1-10)**^**a**^**(*n*** **= 1606)**	Mean 5.70*** (SD 2.40) *n* = 155	Mean 5.65*** (SD 2.32) *n* = 1254	Mean 4.82*** (SD 2.77) *n* = 197
**Do you find it difficult to take care for older patients? (Scale 1-10)**^**b**^**(*n*** **= 1646)**	Mean 3.65* (SD 2.12) *n* = 162	Mean 3.65** (SD 1.98) *n* = 1282	Mean 2.70 (SD 2.05) *n* = 209
**KOP-Q score**	24.95** (SD 2.50) *n* = 164	25.10*** (SD 2.35) *n* = 1294	25.59*** (SD 2.34) *n* = 209

* = correlation 0.10 significant, ** = correlation 0.05 significant, *** = correlation 0.01 significant. ^a^Nurses were asked how they feel about the increase of older patients in the hospital (indicated on a scale from 1 (no problem et al) to 10 (a big problem)). ^b^Nurses were asked whether they find it difficult to care for older patients (indicated on a scale from 0 (very easy) to 10 (very difficult))

## Discussion

The aim of this study was to explore the current knowledge about older patients of registered hospital nurses in the Netherlands and assess whether the level of knowledge is associated with age, level of education, working experience and specialty of ward. Furthermore, registered nurses’ opinions and preferences and how these are related with knowledge levels were explored. The knowledge levels of 36.5% of Dutch nurses fell below the described knowledge levels of registered nurses in literature [15]. This is congruous with prior studies [6, 7, 16]. In a quarterly meeting in April 2018 with representatives of all participating hospitals associated with the RENurse consortium, results were presented and discussed. The group representatives consisted of healthcare professionals, educations and policymakers. They stated that given the complex care needs of older people, it is desirable that knowledge levels of registered nurses are at least within the norm-group scoring range of nurses and results presented were therefore considered alarming.

One important result presented in this study was the wide range in knowledge levels between nurses on both hospital and ward level (Additional file 1). From 79 out 89 wards (88.8%), the range of knowledge levels varied between the norm group student level to the norm group level of nursing specialists (Additional file 1). This implies that an older patient admitted to a ward can receive highly different levels of quality of care on the same day as nurses with different knowledge levels provide care during the various shifts. Complications such as delirium and decubitus can develop quickly [25, 26], with increased risk if nurses are not knowledgeable and act accordingly. It is remarkable that 36.5% of the Dutch nurses who demonstrate scores compared to norm group student levels do not benefit from their colleagues who score better than the norm-group of nurses (i.e., score at nurse specialist level). This result suggests that nurses do not share their knowledge effectively and/or provide peer feedback on a regular basis. When developing future education- and quality improvement programs it is important to take ways of providing peer-feedback effectively into account. Peer feedback is a process to ensure that patient care is delivered according to clinical practice standards, achieving an optimal level of quality [27, 28]. This process supports self-regulation of clinical practice, personal empowerment, and a culture of accountability [29]. As higher knowledge scores were observed in the older, more experiences nurses with higher education levels, and these factors influence the quality of care [30], we believe that this group of nurses can serve as frontrunners, enhancing discussion on their ward and help to facilitate the process of peer feedback.

Secondly, this study demonstrated that most nurses do not find the care for older patients challenging which is in line with literature [6, 7, 9]. This is an important finding taking the low knowledge levels and negative attitudes, with only 12.5% of nurses prefer to care for older patients, into account. If nurses do not find caring for older patients challenging and have other preferences, their internal motivation to follow education and quality improvement programs regarding care for older patients will be low and effects of the education program will be influenced negatively [31]. Confronting individual nurses with their knowledge scores before entering an education program can help in gaining a better insight in one’s own ability’s and knowledge. This insight might provoke and motivate nurses to follow the education program on offer, as they understand there is much to gain for them by doing so [32].

This study did find that nurses who do not prefer taking care of older patients (87.5%) and find it more difficult to care for them demonstrate lower knowledge levels regarding older patients. However, differences in knowledge levels were small. Results do imply that knowledge and attitude constructs such as perceptions and opinions are related and improving knowledge may, therefore, improve attitudes regarding older patients and vice versa. However, to fully understand the relation between these constructs and knowledge, more research is highly recommended.

Some considerations regarding this study should be discussed. For the first time, to our knowledge, the level of knowledge of nurses regarding older patients has been explored and described in a large (national) multicenter context providing insight in the magnitude of knowledge deficits regarding older patients in the Netherlands. To achieve a response rate above 60%, a nurse orientated research coordinator at each hospital site who was familiar with the wards and nurses proved essential [33]. The minimum response rate of 60% is high for an online questionnaire and considered an adequate response rate by most commentators [34]. This, in combination with findings in line from previous research, and the consistent findings between hospitals and wards in our study, makes that we believe that results of this study can be generalized towards other hospitals in the Netherlands. We did not do additional validity and reliability testing of the KOP-Q on this data, which could have decreased the validity of study results. Moreover, a relatively small number of nurses working on the emergency and intensive care department participated. Therefore, it is difficult to generalize the results for these specific groups of nurses, although these results are also in line with previous research [6, 35]. Using an online survey tool which redirected participants who did not provided informed consent to a “thank you” page, makes is difficult to assess who these nurses were leading to insufficient insight in possible selection bias. However, with a sample size of 1743 registered nurses, we feel comfortable that the sample of this study is representative for the Netherlands. By having an online knowledge test, participants have the opportunity to seek information on the internet while answering the questions. Therefore, we assessed the time participants needed to answer all questions and did not see any abnormalities. For this study, patient outcomes were not included. It would be interesting to explore the influence of knowledge levels and attitudes in relation to patient outcomes. Finally, some differences that were found in knowledge levels were small and discussion can exist regarding the clinical relevance. Given the number of nurses included in this study and the variety in knowledge levels on hospital and ward level, the healthcare professionals, education and policymakers of the RENurse consortium share the opinion that these results are of clinical relevance and that nurses’ knowledge regarding older patients should be increased in all participating hospitals.

## Conclusions

Large differences in knowledge regarding older patients exist between Dutch nurses ranging from knowledge levels comparable with nursing students (first and final year) to knowledge levels comparable with nurse specialist. This implies that an older patient admitted to a ward can receive different levels of quality of care on the same day as nurses with different knowledge levels provide care during the various shifts. Moreover, this study shows that most nurses prefer to work with younger age groups over older patients and find the care for older patients easy which is in contrast with the knowledge levels observed. Given these results, addressing the shortcomings of nurses’ knowledge and attitudes towards older patients in educational- and quality improvement programs should be a priority for healthcare professionals, educations and policymakers on a national level.

## Supplementary Information

**Additional file 1.** Number of nurses related to norm group scores on ward and hospital level.

**Additional file 2. **Questions of the knowledge about Older Patient – Quiz [15] and percentage of Dutch hospital nurses (*n* = 1743) who answered the question correct – wrong.

## Data Availability

Data can be shared upon request with J. Dikken (j.dikken@hhs.nl or dikken.j@gmail.com).

## Abbreviations

KOP-Q: Knowledge about Older Patients – Quiz
RENurse: Research and Education in Nursing consortium
S-CVI/ave.: Scale Content Validity Index/average
SD: Standard Deviation
SPSS: Statistical Packages for the Social Sciences
METC: Medical ethics committee
