# Supplementary material for: Hospital nurses’ knowledge regarding older patients: a multicenter study
Source: BMC Nurs. 2021 Aug 4;20:135. doi: 10.1186/s12912-021-00604-4 (PMC8336409; doi:10.1186/s12912-021-00604-4)
Supplement: Supplementary file 1 — Additional file 1. Number of nurses related to norm group scores on ward and hospital level. [file 12912_2021_604_MOESM1_ESM.doc]

Additional file 1: Number of nurses related to norm group scores on ward and hospital level

|  |  |  |  | **Number of nurses scoring within the norm-group knowledge scores on the KOP-Q** | | | |
| --- | --- | --- | --- | --- | --- | --- | --- |
| **Hospital** | **Ward** | **N** | **Range** | **First year** | **Final year** | **Registered** | **Nursing** |
|  |  |  |  | **student** | **student** | **nurses** | **specialist** |
|  |  |  |  | **<21.09** | **21.10-24.25** | **24.26-26.77** | **>26.77** |
| 1 | 1 | 3 | 19-24 | n=1 | n=2 | - | - |
|  | 2 | 13 | 22-29 | - | n=6 | n=4 | n=3 |
|  | 3 | 10 | 20-29 | n=1 | n=2 | n=5 | n=2 |
|  | 4 | 9 | 21-28 | n=2 | n=4 | n=2 | n=1 |
|  | 5 | 21 | 21-30 | n=3 | n=7 | n=3 | n=8 |
| 2 | 1 | 2 | 22-25 | - | n=1 | n=1 | - |
|  | 2 | 27 | 21-28 | n=1 | n=5 | n=8 | n=13 |
|  | 3 | 28 | 20-30 | n=1 | n=6 | n=13 | n=8 |
|  | 4 | 24 | 22-30 | - | n=9 | n=7 | n=8 |
|  | 5 | 31 | 22-30 | - | n=8 | n=11 | n=12 |
|  | 6 | 22 | 23-30 | - | n=3 | n=13 | n=6 |
|  | 7 | 22 | 21-29 | n=1 | n=3 | n=8 | n=10 |
|  | 8 | 21 | 19-30 | n=1 | n=7 | n=6 | n=7 |
|  | 9 | 42 | 19-29 | n=1 | n=8 | n=23 | n=10 |
|  | 10 | 24 | 21-28 | n=2 | n=6 | n=8 | n=8 |
|  | 11 | 15 | 22-29 | - | n=6 | n=5 | n=4 |
| 3 | 1 | 1 | 24 | - | n=1 | - | - |
|  | 2 | 12 | 22-27 | - | n=6 | n=5 | n=1 |
|  | 3 | 20 | 21-30 | n=1 | n=4 | n=3 | n=12 |
|  | 4 | 14 | 20-29 | n=2 | n=6 | n=2 | n=4 |
|  | 5 | 15 | 21-29 | n=2 | n=6 | n=4 | n=3 |
|  | 6 | 16 | 22-29 | - | n=7 | n=5 | n=4 |
|  | 7 | 19 | 22-27 | - | n=12 | n=4 | n=3 |
|  | 8 | 12 | 20-28 | n=5 | n=2 | n=1 | n=4 |
|  | 9 | 16 | 21-27 | n=2 | n=5 | n=5 | n=4 |
|  | 10 | 7 | 22-26 | - | n=3 | n=4 | - |
|  | 11 | 14 | 20-29 | n=1 | n=3 | n=6 | n=4 |
|  | 12 | 15 | 20-30 | n=1 | n=4 | n=4 | n=6 |
|  | 13 | 16 | 15-28 | n=1 | n=6 | n=6 | n=3 |
|  | 14 | 10 | 20-26 | n=1 | n=3 | n=6 | - |
|  | 15 | 13 | 20-28 | n=4 | n=4 | n=1 | n=4 |
|  | 16 | 15 | 15-29 | n=1 | n=3 | n=6 | n=5 |
|  | 17 | 12 | 17-29 | n=2 | n=4 | n=2 | n=4 |
|  | 18 | 22 | 20-28 | n=1 | n=7 | n=8 | n=6 |
|  | 19 | 16 | 21-30 | n=1 | n=1 | n=6 | n=8 |
|  | 20 | 39 | 21-28 | n=1 | n=10 | n=18 | n=10 |
|  | 21 | 12 | 21-29 | n=2 | n=3 | n=2 | n=5 |
| 4 | 1 | 1 | 24 | - | n=1 | - | - |
|  | 2 | 18 | 21-29 | n=1 | n=5 | n=5 | n=7 |
|  | 3 | 19 | 19-28 | n=3 | n=6 | n=6 | n=4 |
|  | 4 | 20 | 21-29 | n=2 | n=6 | n=7 | n=5 |

| **Hospital** | **Ward** | **N** | **Range** | **First year** | **Final year** | **Registered** | **Nursing** |
| --- | --- | --- | --- | --- | --- | --- | --- |
|  |  |  |  | **student** | **student** | **nurses** | **specialist** |
|  |  |  |  | **<21.09** | **21.10-24.25** | **24.26-26.77** | **>26.77** |
|  | 5 | 24 | 22-29 | - | n=4 | n=6 | n=14 |
| 5 | 1 | 20 | 19-29 | n=2 | n=5 | n=6 | n=7 |
|  | 2 | 23 | 20-29 | n=2 | n=7 | n=7 | n=7 |
|  | 3 | 19 | 21-28 | n=3 | n=11 | n=2 | n=3 |
|  | 4 | 10 | 21-28 | n=1 | n=2 | n=4 | n=3 |
| 6 | 1 | 2 | 26/28 | - | - | n=1 | n=1 |
|  | 2 | 42 | 20-29 | n=3 | n=11 | n=15 | n=13 |
|  | 3 | 23 | 20-30 | n=2 | n=5 | n=7 | n=9 |
|  | 4 | 42 | 20-29 | n=1 | n=15 | n=18 | n=8 |
|  | 5 | 17 | 21-28 | n=1 | n=7 | n=4 | n=5 |
|  | 6 | 36 | 20-30 | n=2 | n=5 | n=8 | n=21 |
|  | 7 | 27 | 21-29 | n=2 | n=11 | n=8 | n=6 |
|  | 8 | 34 | 22-29 | - | n=13 | n=7 | n=14 |
| 7 | 1 | 2 | 25 | - | - | n=2 | - |
|  | 2 | 16 | 20-28 | n=1 | n=4 | n=4 | n=7 |
|  | 3 | 14 | 22-26 | - | n=10 | n=4 | - |
|  | 4 | 23 | 19-28 | n=2 | n=4 | n=9 | n=8 |
|  | 5 | 26 | 20-29 | n=3 | n=6 | n=7 | n=10 |
|  | 6 | 12 | 22-29 | - | n=6 | n=3 | n=3 |
|  | 7 | 12 | 22-30 | - | n=4 | n=4 | n=4 |
|  | 8 | 19 | 21-29 | n=1 | n=7 | n=7 | n=4 |
|  | 9 | 17 | 21-29 | n=1 | n=3 | n=5 | n=8 |
|  | 10 | 15 | 23-29 | - | n=5 | n=6 | n=4 |
|  | 11 | 16 | 17-28 | n=3 | n=9 | n=2 | n=2 |
|  | 12 | 31 | 21-27 | n=3 | n=7 | n=15 | n=6 |
| 8 | 1 | 9 | 22-28 | - | n=3 | n=3 | n=3 |
|  | 2 | 28 | 17-30 | n=4 | n=8 | n=8 | n=8 |
|  | 3 | 19 | 17-29 | n=2 | n=5 | n=7 | n=5 |
|  | 4 | 15 | 22-29 | - | n=4 | n=6 | n=5 |
|  | 5 | 16 | 15-27 | n=2 | n=1 | n=12 | n=1 |
|  | 6 | 14 | 20-28 | - | n=4 | n=8 | n=2 |
| 9 | 1 | 6 | 18-29 | n=1 | n=1 | n=2 | n=2 |
|  | 2 | 14 | 18-29 | n=3 | n=7 | n=3 | n=1 |
|  | 3 | 8 | 20-30 | n=1 | n=2 | n=2 | n=3 |
|  | 4 | 14 | 20-28 | n=1 | n=8 | n=2 | n=3 |
|  | 5 | 17 | 20-29 | n=3 | n=5 | n=4 | n=5 |
|  | 6 | 19 | 19-29 | n=1 | n=5 | n=9 | n=4 |
| 10 | 1 | 2 | 26 | - | - | n=2 | - |
|  | 2 | 11 | 18-29 | n=1 | n=2 | n=7 | n=1 |
|  | 3 | 21 | 20-29 | n=2 | n=1 | n=6 | n=12 |
|  | 4 | 22 | 18-28 | n=1 | n=6 | n=9 | n=6 |
|  | 5 | 19 | 20-29 | n=1 | n=5 | n=4 | n=9 |
|  | 6 | 57 | 18-29 | n=2 | n=15 | n=23 | n=17 |

| **Hospital** | **Ward** | **N** | **Range** | **First year** | **Final year** | **Registered** | **Nursing** |
| --- | --- | --- | --- | --- | --- | --- | --- |
|  |  |  |  | **student** | **student** | **nurses** | **specialist** |
|  |  |  |  | **<21.09** | **21.10-24.25** | **24.26-26.77** | **>26.77** |
| 10 | 7 | 39 | 22-30 | - | n=13 | n=10 | n=16 |
|  | 8 | 67 | 20-30 | n=5 | n=24 | n=22 | n=16 |
|  | 9 | 51 | 18-29 | n=3 | n=15 | n=15 | n=18 |
|  | 10 | 34 | 20-30 | n=1 | n=10 | n=6 | n=17 |
|  | 11 | 31 | 22-29 | - | n=12 | n=11 | n=8 |
